# Pay to Play or Pay to Lose? The Impact of In-game Spending in Digital Games on Gambling Problems

**DOI:** 10.1007/s10899-025-10453-4

**Published:** 2025-11-04

**Authors:** Ilkka Vuorinen, Iina Savolainen, Atte Oksanen

**Affiliations:** Emerging Technologies Lab, Faculty of Social Sciences, Tampere University, Tampere, FI-33014 Finland

**Keywords:** Gambling, Gaming, Microtransactions, Longitudinal research, Mental health, Alcohol use

## Abstract

During the mid-2010s, microtransactions within digital games became more common, raising concerns that the gambling-like features in these games would contribute to increases in gambling problems. However, studies on the topic have been mostly cross-sectional. To fill this gap, this study uses longitudinal nationwide survey data collected from 949 adult Finnish participants every six months from the spring of 2021 to the fall of 2024 to investigate the impact of microtransactions on gambling problems—measured using the Problem Gambling Severity Index (PGSI)—over time. Gambling participation, mental health issues, and alcohol use were used as control variables, and age and gender were used as background variables. The results showed that although microtransactions had significant positive fixed and random effects on gambling problems, the effects disappeared when gambling participation was added to the models. Therefore, the effect of microtransactions on gambling problems may only be indirect. This implies that the main regulatory focus should be on actual gambling participation, whether or not it is encouraged by the purchasable content in some digital games.

Digital games have changed significantly in recent decades. As internet connections have become faster and more widely available, an increasing number of multiplayer games can be played online with virtually any other players who are online. Even when the game is intended to be played alone, platforms such as Steam (developed by Valve Corporation) allow players to add social elements to their gaming by sharing achievements or pictures of their gameplay with the community. While gaming has become more social in these online environments, not all developments have been for mere entertainment. To explore one potentially harmful development, the convergence between gambling and gaming, this study focuses on microtransactions in digital games and their impact on gambling problems.

Similarities between gambling and digital games had already been noted in the early 1990s (Griffiths, 1991), and the presence of gambling on digital media platforms has raised concerns, especially regarding its influence on adolescents (King et al., 2010). Although all kinds of games can be played for reasons such as enjoyment and challenge, the gambling industry has been motivated to soften attitudes toward gambling by referring to it as gaming. However, gambling is defined as wagering objects of value for uncertain outcomes (Derevensky & Griffiths, 2019). This makes gambling a riskier form of play, as financial, health, and psychosocial problems often impact individuals who gamble and the people close to them, burdening society on many levels (Hautamäki et al., 2025). Longitudinal research has shown that excessive gaming can also lead to issues with health and wellbeing (Amendola et al., 2025), but the financial risks are typically not as much of a concern.

In addition to monetary losses and indebtedness, problematic gambling has been linked to family disruptions, reduced work performance, dishonest or even criminal activities, and several mental health issues such as mood and anxiety disorders (Sulkunen et al., 2019). Worse still, suicidality is higher among individuals with gambling problems (Kristensen et al., 2024), reflecting the despair that excessive gambling losses can create. Both the World Health Organization (2019) and the American Psychiatric Association (2013) have placed gambling disorder in the same category as substance use disorders, emphasizing the behavioral similarities between these activities and the need for treatment that some gamblers require.

Although digital games are more entertainment-based than gambling, the gaming industry also needs ways to generate revenue. In addition to one-time purchased full games, additional purchases within the game (also known as microtransactions) have become an increasingly common way to pay for games (Zendle et al., 2020). These purchases range from cosmetic upgrades to new content, in-game currency, or changes in the pace of the game, and these have yielded mixed reactions from the gamers (Švelch, 2017). A game may be initially free to play, but it can include advertising or other disruptions that try to exploit player impatience and encourage players to perform microtransactions to advance faster in the game (Evans, 2016; Turner & Shi, 2024). In addition to obtaining unobstructed gameplay, social and price-related motives can increase the chances of players making microtransactions in games (Hamari et al., 2017).

Microtransactions can also be seen as a form of gamblification when games involve gambling-like elements, such as the possibility of using in-game items for betting on external sites and purchasing in-game containers (loot boxes) for a chance to win rare items (Hing et al., 2022; King & Delfabbro, 2020). Loot boxes in particular have raised concerns regarding their characteristics, as they resemble gambling to varying extents, depending on the game (Drummond & Sauer, 2018).

The extent to which microtransactions resemble gambling has been widely debated, since they are distinct from traditional gambling. One argument against classifying microtransactions as gambling is that in-game items typically do not hold monetary value outside the game and cannot therefore be considered equivalent to gambling rewards (Abarbanel, 2018; Brooks & Clark, 2023; Griffiths, 2018). This view has been increasingly challenged, as a growing body of research suggests that such chance-based in-game mechanisms are structurally equivalent to wagering and they share psychological features that appeal to players (Gibson et al., 2022, 2025). These debates have also been reflected at the policy level, with many countries now considering microtransactions such as loot boxes to be a prohibitable form of monetization (Xiao et al., 2022; Xiao, 2024). Furthermore, microtransactions are consistently linked to problematic gaming and gambling behaviors (Raneri et al., 2022), although studies on this topic have mostly been cross-sectional, and longitudinal research is needed to better understand these relationships.

This study continues this investigation by examining the effect of microtransactions on gambling problems in a longitudinal setting. Based on the available literature, it is expected that microtransactions increase gambling problems; however, our aim is to elaborate this association further, controlling for mental health issues, alcohol use, and gambling participation. Although being an active gambler is likely to predict gambling problems, we intend to determine whether microtransactions still have predictive power on these problems when gambling participation is taken into account.

## Methods

### Participants

A longitudinal survey study on a representative sample of participants from mainland Finland was conducted in six-month intervals between April–May 2021 (T1) and October–November 2024 (T8). Data collection was carried out by Norstat on behalf of the research team at all time points. At T1, 1,530 participants between the ages of 18 and 75 took part in the survey. The response rate was 31.32% in this first wave. Participants were recontacted every six months, and approximately 46% of them responded to all follow-up surveys. Attrition was more pronounced among younger participants during the follow-up, leading to the sample composition weighting more heavily toward middle-aged individuals. At Time 1, 29.15% of participants were under 35 years old whereas only 13.84–17.56% were under 35 years old in later survey time points (T5, Spring 2023 to T8, Fall 2024). About half of the participants identified as men and the other half as women at each time point, whereas at T1, four participants (0.26%) marked their gender as “other” or alternated between the binary gender identities at the next time points.

The Ethics Committee of the Tampere Region approved the study before data collection began. Participants were informed of the purpose of the study, and they consented to participate by responding to the full survey. At each time point, the estimated duration for the full survey was 15 min, but response times varied. After the research team received the fully anonymized data from Norstat, we checked for biased response patterns based on our preregistered protocol, removing some participants at each time point.

For the purposes of this study, we only included participants who had played digital games. The remaining number of participants in each wave was 949 (T1), 709 (T2), 653 (T3), 569 (T4), 536 (T5), 504 (T6), 516 (T7), and 461 (T8). In this final dataset, 52.07% identified as men, and the mean age ranged from 42.49 years (T1) to 48.90 years (T8).

### Measures

Gambling problems were measured using the Problem Gambling Severity Index (PGSI), which is commonly used in population-wide surveys both globally (Grigsby, 2020) and in Finland (Salonen et al., 2020). Its nine items cover gambling problems based on the diagnostic criteria of disordered gambling on a scale of 0 (*never*), 1 (*sometimes*), 2 (*most of the time*), and 3 (*nearly always*), which are summed to a count variable with a score ranging from 0 to 27. The mean scores for the scale ranged from 1.20 to 1.72, which means that its distribution was highly skewed to the right. The reliability of the scale, measured at T1, was 0.94.

To measure microtransaction spending, the participants were asked how they mainly paid for their gaming; they could choose either 0 (*no*) or 1 (*yes*) in response to this question. The number of participants who reported paying with microtransactions was 90 at T1 and 47 at T8, with 46 at T7 being the lowest number. This means that around 10% of participants had paid with microtransactions in each wave. The lowest percentage was 8.91% at T7, and the highest was 11.07% at T4. The measure was used as a categorical variable in the analysis. 

We asked participants to evaluate how often they had recently gambled on different platforms, including online casinos, online poker, table games, betting, slot machines, lottery, scratch cards, private betting, and cue-sport gambling. The options were 0 (*never*), 1 (*less than once a month*), 2 (*monthly*), 3 (*weekly*), 4 (*once per day*), and 5 (*multiple times per day*). These items were combined into a sum variable ranging from 0 to 45 to control for gambling participation. In line with the PGSI, the mean scores indicated high skewness for the distribution, ranging from 5.56 at T1 to 4.54 at T8. The reliability of this sum variable was 0.86 at T1.

The five-item Mental Health Inventory (MHI-5; Berwick et al., 1991) is a commonly used measure of general mental health in Finnish public health studies (Salonen et al., 2020). Three of the five items measured nervousness and depressed mood, whereas two items measured general positive affect and had to be inverted in the formation of the scale. Participants were asked to evaluate these states on a scale from 1 (*none of the time*) to 6 (*all of the time*), which yielded scores ranging from 5 to 30 when all the items were summed together and used for the analyses as a continuous measure. The mean scores for the variable stayed relatively stable during the measuring period, with the lowest score being 12.41 at T6 and the highest being 13.05 at T4. The reliability of this scale was 0.89 at T1.

Alcohol use was measured with the consumption-focused items of the Alcohol Use Disorders Identification Test (AUDIT-C; Bush et al., 1998). Like the previous measures, it is also often used in Finnish public health studies (Salonen et al., 2020). The AUDIT-C consists of three items. In the first one, participants are asked to evaluate how often they drink alcohol from 1 (*never*) to 4 (*four or more times a week*). The second item measured the dosage of alcohol consumption from 0 (*I never drink alcohol*) to 5 (*10 or more*). The third item measured how often participants consumed six or more units of alcohol from 0 (*never*) to 4 (*daily or almost daily*). Given the risk-point system of the measure, we used it as a continuous variable in the analysis. The mean scores ranged from 3.79 at T1 to 3.52 at T8. The reliability of this scale was 0.80 at T1.

We used gender and age as background variables. Gender was formed into a binary variable where 1 = man and 0 = woman or other gender, whereas age was left in continuous form. The descriptive statistics for each measure are shown in Table 1.

**Table 1:** Descriptive statistics of included variables across timepoints (T1–T8)

Continuous variables	Range	M_T1_ (SD)	M_T2_ (SD)	M_T3_ (SD)	M_T4_ (SD)	M_T5_ (SD)	M_T6_ (SD)	M_T7_ (SD)	M_T8_ (SD)
PGSI scores	0–27	1.72 (3.82)	1.55 (3.58)	1.54 (3.74)	1.38 (3.31)	1.27 (3.05)	1.20 (2.96)	1.24 (3.04)	1.29 (3.30)
Gambling participation	0–45	5.56 (4.83)	5.66 (5.04)	5.33 (4.89)	5.21 (4.86)	4.95 (4.51)	4.95 (4.33)	4.69 (4.69)	4.54 (4.65)
MHI-5	5–30	12.98 (4.72)	12.87 (4.53)	12.89 (4.51)	13.05 (4.57)	12.50 (4.59)	12.41 (4.45)	12.68 (4.69)	12.63 (4.56)
AUDIT-C	0–12	3.79 (2.64)	3.71 (2.72)	3.70 (2.68)	3.58 (2.69)	3.58 (2.69)	3.59 (2.68)	3.59 (2.63)	3.52 (2.76)
Age	18–75 (at T1)	40.80 (15.18)	42.70 (14.94)	43.52 (15.14)	44.59 (14.86)	46.52 (14.64)	47.97 (14.68)	48.37 (14.71)	48.56 (14.69)
Categorical variables		***n***_**T1**_ **(%)**	***n***_**T2**_ **(%)**	***n***_**T3**_ **(%)**	***n***_**T4**_ **(%)**	***n***_**T5**_ **(%)**	***n***_**T6**_ **(%)**	***n***_**T7**_ **(%)**	***n***_**T8**_ **(%)**
Microtransactions	0–1	90 (9.48)	68 (9.59)	64 (9.80)	63 (11.07)	58 (10.82)	49 (9.72)	46 (8.91)	47 (10.20)
Male gender	0–1	508 (53.53)	388 (54.72)	341 (52.22)	303 (53.25)	291 (54.29)	272 (53.97)	278 (53.88)	250 (54.23)

Mean (M) scores and standard deviations (SD) are reported for continuous variables and frequencies (*n*) and percentages (%) for categorical variables, including those who had paid mainly with microtransactions for their gaming and those who reported male gender

### Statistical Analyses

All analyses were conducted with StataNow/MP version 18.5 by StataCorp LLC. Following Hayes and Coutts (2020), we used McDonald’s ω (using the *omegacoef* command in Stata) to measure the reliability of the scales. The PGSI was the dependent count variable in the models, with microtransactions being the main independent variable. Other independent variables were added to the models to see how they affect the impact of microtransactions on the PGSI. In our main models, we used negative binomial regression to examine the fixed and random effects of the independent variables on PGSI scores over time. This was conducted utilizing *xtnbreg* command with the *fe* and *re* specifications. With highly skewed count variables such as the PGSI, negative binomial family and log link functions are typical specifications for obtaining more precise model estimates (Baggio et al., 2018). To aid interpretation, the results were exponentiated to incidence rate ratios (IRR) which reflect multiplicative change in expected PGSI scores for each one-unit change in the independent variables. Of these two, fixed effects (FE) models are more regularly used as they control for all between-person variance—including age and gender—to avoid heterogeneity bias, focusing only on within-person estimates, whereas random effects (RE) models offer more generalizability but the exogeneity assumption behind it is often violated (Bell & Jones, 2015). We also checked whether gambling activity interacts with the effect of microtransactions on PGSI scores.

## Results

Based on the correlations at T1 (see Table 2), each independent variable had a statistically significant connection to PGSI scores. Understandably, being an active gambler correlates strongly with gambling problems (0.57; *p* <.001), but mental health issues and alcohol use also have relatively high correlations with PGSI scores (0.21; *p* <.001 for both variables). Microtransactions had only weak correlations at T1, ranging from 0.09 (*p* <.01) with PGSI scores and mental health issues to 0.11 (*p* <.001) with gambling participation and − 0.14 (*p* <.001) with age. It is also noteworthy that gambling participation had a 0.30 (*p* <.001) correlation with alcohol use and 0.22 (*p* <.001) correlation with gender, with men being more likely to gamble.

**Table 2:** Correlation matrix, variables at T1

	1.	2.	3.	4.	5.	6.	7.
1. PGSI	1						
2. Microtransactions	0.09**	1					
3. Gambling participation	0.57***	0.11***	1				
4. MHI-5	0.21***	0.09**	0.08*	1			
5. AUDIT-C	0.21***	0.03	0.30***	0.11***	1		
6. Age	− 0.09**	− 0.14***	− 0.02	− 0.26***	− 0.06	1	
7. Male gender	0.11***	0.06	0.22***	− 0.11***	0.22***	0.00	1

* *p* <.05; ** *p* <.01; *** *p* <.001

Longitudinal models (Table 3) confirmed much of what can already be seen in the correlation matrix. Microtransactions had a significant independent fixed effect (1.28; *p* <.001) and random effect (1.36; *p* <.001) on gambling problems, meaning that microtransaction payments predicted a 28% increase in gambling problem scores in the fixed effects model, and by 36% increase in the random effects model. However, this significance disappeared when gambling participation was added to the models, which in turn predicted a 7–8% increase in the PGSI for every one-unit increase. Furthermore, mental health issues and alcohol use had significant fixed and random effects on gambling problems, but the random effect of gender was nonsignificant, and age had a negligible random effect on PGSI scores. The final model group shows that there was a significant but small negative interaction (0.92; *p* <.05) between the random effects of microtransactions and gambling participation, indicating that microtransactions are less strongly associated with gambling problems when people are more active gamblers. The interaction in the fixed effects model was not significant.

**Table 3:** Fixed effects (FE) and random effects (RE) models showing the incidence rate ratios of independent variables on PGSI

	*Model group 1*					*Model group 2*				*Model group 3*			
	FE	(SD)		RE	(SD)	FE	(SD)	RE	(SD)	FE	(SD)	RE	(SD)
Microtransactions	1.28 ***		(0.09)	1.36 ***	(0.09)	1.10	(0.07)	1.12	(0.07)	1.28 *	(0.16)	1.32 **	(0.12)
Gambling participation	-			-		1.07 ***	(0.00)	1.08 ***	(0.00)	1.38^1^ ***	(0.03)	1.48^1^ ***	(0.03)
MHI-5	-			-		1.03 ***	(0.01)	1.04 ***	(0.01)	1.03 ***	(0.01)	1.04 ***	(0.01)
AUDIT-C	-			-		1.03 *	(0.01)	1.04 **	(0.01)	1.03 *	(0.01)	1.04 **	(0.01)
Male gender	-			-		-		1.16	(0.14)	-		1.16	(0.14)
Age	-			-		-		0.99*	(0.00)	-		0.99 *	(0.00)
*Interaction*: Microtransactions X gambling participation	-			-		-		-		0.95	(0.03)	0.92 *	(0.03)
Wald χ^2^ test	11.19 ***			20.09 ***		298.55 ***		509.75 ***		299.66 ***		513.74 ***	
Hausman test	6.54 *					649.85 ***				793.89 ***			

* *p* <.05; ** *p* <.01; *** *p* <.001

^1^ Standardized variable

FEs have only 448 participants after dropping participants with only one observation per participant or all zero outcomes. REs have 1,208 participants

Standard errors in parentheses

## Discussion

This longitudinal study investigated the fixed effects and random effects of microtransactions on gambling problems, accounting for other possible factors. Our findings show that while microtransactions can have a significant impact on gambling problems among the Finnish adult population who have played digital games at least sometimes, the statistical significance of this effect seems to disappear when other factors like gambling participation are taken into consideration. However, interaction models suggest that the predictive power of microtransactions is not entirely diminished when gambling participation is controlled for, but rather may be contingent upon their interaction, highlighting the complexity of their relationship. This interaction seems to be present between the random effects of microtransactions and gambling participation, although Hausman test indicates that the assumptions behind the model might not hold and should be interpreted with caution. 

Previous studies on microtransactions have raised concerns about their association with gambling, especially regarding loot boxes (Drummond & Sauer, 2018; Raneri et al., 2022). Since these studies tend to be cross-sectional, conclusions regarding causality have been difficult to draw. They have also mainly focused on young people. Our study thus contributes to the previous research on microtransactions by bringing a longitudinal perspective on the matter, investigating an adult population mainly consisting of participants older than 25 years of age, and adjusting for gambling participation. The results we obtained from the models suggest that the effects of microtransactions on gambling problems are better explained by frequency of gambling involvement, which is linked to many serious personal and social issues (Hautamäki et al., 2025; Sulkunen et al., 2019). Furthermore, gambling and microtransactions can be competing spending options for individuals, so prioritizing one leaves less money for the other activity. This could be explored in more detail in further studies. In our sample, spending behavior may also reflect patterns more typical of gambling rather than microtransactions. Younger players, who tend to engage more frequently with microtransactions in games (Gibson et al., 2022), may experience these spending mechanisms differently due to their experiences with these digital gaming environments. 

Although excessive gaming can conflict with obligations in life and raise issues regarding health and wellbeing (Amendola et al., 2025; Coyne et al., 2012), digital games do not necessarily involve frequent use of money and are typically played for entertainment. Therefore, incorporating gambling-like elements with microtransactions to digital games seems like a problematic revenue model. The risk of problematic gambling has been found to increase with higher microtransaction expenditure (Raneri et al., 2022), and game developers seem to profit from people who are at risk of developing gambling problems (Close et al., 2021). While our results imply that gambling participation matters more regarding gambling problems, gambling-like elements in digital games may still encourage at-risk individuals to gamble. Since microtransactions can resemble gambling in the way they are presented with gambling-like sounds and visual effects in digital games (Gibson et al., 2022), it is possible that microtransactions are indirectly linked to gambling problems through gambling participation.

Although the longitudinal design of this study enhances the understanding of these associations, there are some important limitations to point out. Most importantly, because people participated in our survey based on their interest, the data collection could not be fully randomized. Some of the participants also dropped out when new survey waves were collected. Especially the loss of younger participants over time may have influenced the findings, as engagement with microtransactions has previously been found to be more common among younger individuals, who may also experience these game mechanisms differently due to their greater generational exposure to such gaming features. Still, the data represented the Finnish population well. Another limitation of our study is the low number of participants who mainly paid for their gaming with microtransactions. It is possible that more participants had spent money on microtransactions but did so more occasionally, or even played digital games less frequently, leaving this payment method unchecked. Having a larger group of those who have paid for in-game content with microtransactions would perhaps provide more robust estimates of the already important findings of this study.

The results of this study are indicative of the general population, and they imply that microtransactions in digital games do not increase the risk of gambling problems directly when gambling participation is taken into account. Despite this, it would be worthwhile to regulate gambling-like elements in video games by following measures listed by, for example, King and Delfabbro (2020). Purchasing in-game content can bring more exciting features to digital games, but these processes need to be transparent and ethical. The costs of gambling to individuals and the people around them are already high, and it would be best for digital game developers to follow monetization strategies that minimize such risks.
